# Novel filter-press single-step cleanup approach facilitated rapid screening and accurate quantification of 112 veterinary drugs in aquatic products

**DOI:** 10.1016/j.fochx.2023.100846

**Published:** 2023-08-19

**Authors:** Jincheng Li, Ruidong Zhou, Guangxin Yang, Essy Kouadio Fodjo, Tengwang Feng, Huiwu Sun, Dongmei Huang, Cong Kong, Huan Liu

**Affiliations:** a Key Laboratory of Control of Quality and Safety for Aquatic Products, Ministry of Agriculture and Rural Affairs, Chinese Academy of Fishery Sciences, Beijing 100141, PR China; b College of Fisheries and Life Science, Shanghai Ocean University, Shanghai 201306, PR China; c Key Laboratory of East China Sea Fishery Resources Exploitation, Ministry of Agriculture and Rural Affairs, East China Sea Fisheries Research Institute, Chinese Academy of Fishery Sciences, Shanghai 200090, China; d Laboratory of Constitution and Reaction of Matter (Physical Chemistry), Université Felix Houphou-et-Boigny, 22 BP 582 Abidjan 22, Cote d’Ivoire

**Keywords:** Drug residues, Sample pretreatment, Batch purification, UHPLC-Q-Orbitrap-HRMS, Fish

## Abstract

In this study, a novel filter-press cleanup column was developed as a single-step cleanup approach for the rapid screening and quantification of 112 veterinary drugs in fish samples. Fish muscle samples were extracted with acetonitrile and ethyl acetate, sequentially. After concentration and reconstitution, *N*-propylethylenediamine (PSA) sorbent, packed in filter-press column, allows rapid single-step cleanup operation, while UHPLC-Q-Orbitrap-HRMS provides high-precision mass information in multi-residue screening. Under optimum settings, the detection and quantification limits were validated at 0.5 and 2.0 μg·kg^−1^, for all analytes, respectively. The ranges of recoveries were from 35.3 to 138.4%. Most of these target analytes (82%) could be measured with recoveries between 60 and 130%, and intra-day RSDs ranging from 1.9 to 26.1%. This method was further applied to evaluate the residual of veterinary drugs in fish samples from four cities in China, and results demonstrated its practicability for multi-residue monitoring veterinary residues for food safety administration.

## Highlights

• Rapid screening and quantification of 112 veterinary drugs in fish samples by UHPLC-Q-Orbitrap-HRMS is demonstrated.
• The results accuracy could be ascribed to the effective sample preparation and the high-resolution of HRMS.
• A novel filter-press single-step clean-up method was proposed based on r-DSPE.
• Multiple samples could be processed in parallel manually using this method.
• 87% of these target analytes can be measured with the recoveries of 60 to 130%.

## Graphical abstract

**Figure:**
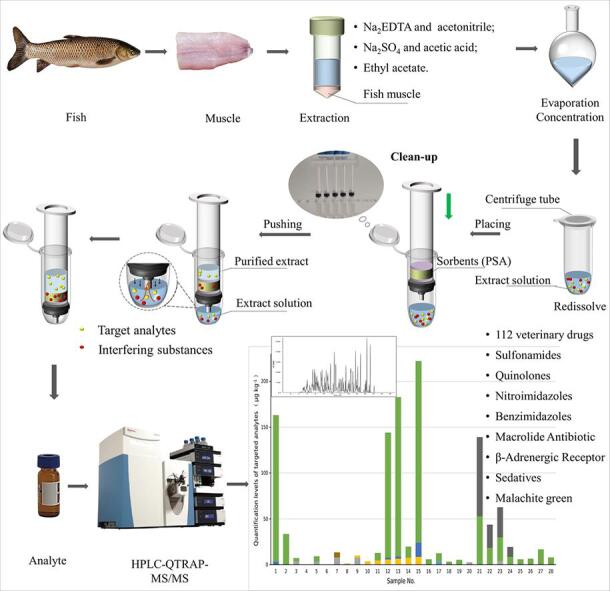

## 1 Introduction

Aquatic products are rich in nutrition and favored by consumers in China. Most of these aquatic products are obtained by artificial farming methods. In order to control and treat bacterial infections and other diseases, veterinary drugs are often used. The increasing risks to human health have been confirmed generated by the widespread use of veterinary medicines in aquaculture. To ensure the safety of aquatic products, more and more drug residues such as sulfonamides, quinolones, tetracyclines in aquatic products have been subject to routinely monitoring in China. Efficient methods are needed for multi-residue analyzing veterinary drugs in aquatic products, but the complexity of aquatic product matrices constitutes challenges in their analysis (He et al., 2019).

So far, various analytical methods for veterinary drugs detection have been developed. However, the reported methods of veterinary drugs are generally complex. For example, high-performance liquid chromatography-tandem mass spectrometry (HPLC-MS) based methods, liquid–solid extraction (Zhang et al., 2020) by large-volume organic solvent or solid phase extraction (Li et al., 2019), and other sample pretreatment approaches are frequently used. Researchers have been paying more attention to the analysis of complex systems, and numerous efficient methods for sample preparation have been established, including magnetic solid phase extraction (Wei et al., 2019, Molaei et al., 2020), magnetic-assisted dispersive solid phase extraction (Kaw et al., 2021), QuEChERS (effective, easy, quick, cheap, rugged, and safe) approach (Qiu et al., 2017), etc.

Extraction of aquatic products is generally performed by using organic solvents such as acetonitrile (Shin et al., 2021), and then cleaned by solid phase extraction column (SPE) (Liao et al., 2015), reversed-dispersive solid-phase extraction (r-DSPE) (Shin et al., 2018) or other methods (Molaei et al., 2020). After concentrating and redissolving, quantitative analysis is conducted by HPLC-MS (Maciążek-Jurczyk et al., 2020), ultrahigh performance liquid chromatography quadrupole Orbitrap high-resolution mass spectrometry (UHPLC-Q-Orbitrap-HRMS) (Wang et al., 2021) or other detectors. The cleanup process is a critical and time consuming step. For instance, purification processes of solid phase extraction column consists of four steps (Shen et al., 2021): activation, sample loading, wash and elution. The purification process of conventional r-DSPE cleanup method consists of three steps (Emami et al., 2022): vortex mixing the sorbents and crude acetonitrile extract to retain interfering substances (such as lipids, fatty acids, etc) due to the electrostatic interaction and other hydrophobic interactions, centrifugation and then filtering the supernatant. The operation is time-consuming and complicated. It has become a common understanding for researchers to develop simpler cleanup methods for sample extracts, especially miniaturized and automatic sample pretreatment devices.

In the past few years, the r-DSPE cleanup method combined with QuEChERS approach has been developed with further modifications to enhance its efficiency (Dai et al., 2023). Many syringe-type r-DSPE cleanup methods were reported (Han et al., 2018, Mu et al., 2021). r-DSPE sorbent is packed into a syringe such as multi-walled carbon nanotubes, *N*-propylethylenediamine (PSA), octadecyl bonded silica gel particles (C_18_) and anhydrous magnesium sulfate. After adding the extract, it can be passed through the r-DSPE layer by pushing or pulling the syringe plunger. r-DSPE sorbent can absorb interfering substances in the matrix selectively. The single-step QuEChERS (sin-QuEChERS) cleanup method is a novel r-DSPE technique (Huang et al., 2021). The Sin-QuEChERS cleanup column is packed with r-DSPE sorbent, and then inserted into a 50 mL centrifuge tube containing the extract for purification. The extract can be purified in one step by downward pressing the column. This approach has been successfully used for analysis of pesticide residues in tea (Huang et al., 2021), chives (Li et al., 2020), etc.

**Fig. 1:**
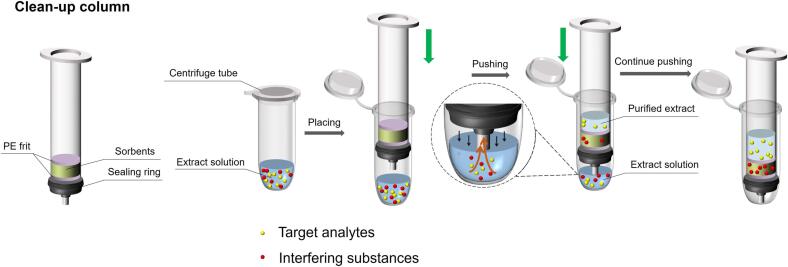
Workflow of the filter-press cleanup column.

Herein, we present a filter-press cleanup column based on the Sin-QuEChERS cleanup column. The filter-press cleanup column structure is presented in Fig. 1. Suitable r-DSPE sorbent packed in the column is used to absorb interfering substances in the matrix. A rubber O-sealing ring is fixed around the outlet of the cartridge. After the extract is added into a centrifuge tube, filter-press cleanup column can be inserted into the centrifuge tube. Pushing of the column promotes the passing of the extract solution through the sorbent’s layer from the bottom to the top. The presented filter-press cleanup column combined with QuEChERS extraction method was applied to rapid single-step cleanup operation of fish muscle tissues.

According to the regulation and routine monitoring of aquatic products by the Chinese administration, 112 veterinary drugs were selected in our study. After single-step cleanup by the filter-press cleanup column, UHPLC-Q-Orbitrap-HRMS approach was used for rapid screening as well as quantification of the 112 veterinary drugs. Furthermore, this study evaluated veterinary residues in fish samples taken from four different provinces in China.

## 2 Experimental section

### 2.1 Reagents and materials

Promethazine was obtained from Dr. Ehrenstorfer (GmbH, Germany). All the other 111 standard veterinary drugs were procured from Tianjin Alta Scientific Co., Ltd (Tianjin, China). Acetic acid and anhydrous sodium sulfate were obtained from Sinopharm Chemical Reagent Co., Ltd (Shanghai, China). Ammonium acetate was bought from Thermo Fisher Scientific Inc. (MA, USA). Formic acid was procured from Aladdin Biochemical Technology Co., Ltd (Shanghai, USA). Methanol, acetonitrile and ethyl acetate were supplied by J. T. Baker (Philipsburg, NJ, USA). PSA (40–63 μm, 60 Å), C_18_, graphitic carbon black (GCB) and alumina-N were provided by Bestown (Beijing, China). Polystyrene-divinyl benzene sorbents (PS-DVB) were bought from Anpel Scientific Instrument Co. Ltd (Shanghai, China).

Mixture standard solutions of 112 standard veterinary drugs were obtained from standard stock solutions via serial dilutions and kept at −20 °C for use.

### 2.2 Construction of filter-press cleanup column

In Fig. 1, the filter-press cleanup column was prepared as follows:

(i) A polyethylene frit was positioned in the bottom of SPE.
(ii) The SPE was packed with sufficient amount of r-DSPE sorbents.
(iii) Another frit was positioned on the r-DSPE sorbent layer.
(iv) A rubber O-sealing ring was placed at the column outlet, and a filter-press cleanup column was constructed.

### 2.3 Sample collection and preparation

Fish samples (live) were obtained from freshwater aquaculture farms in four different cities (Shanghai, Luoyang, Zhengzhou, and Kaifeng) in China. The live fish were immediately sacrificed by a blow to the head, transferred in a sealed plastic bag, and instantly frozen in refrigerator at −18 °C. In the lab, edible fish fillets were detached from the fish body, homogenized, and kept at −18 °C for subsequent analyses.

Extraction was adopted according to our previous work with some modifications (Kong et al., 2018). A sample (5.0 g) was placed into a centrifuge tube (50 mL). 1.0 mL Na_2_EDTA solution (0.1 mol/L) was added and supplemented with 10 mL of acetonitrile. After vortexing at 2500 rpm for 10 min, 5.0 g of anhydrous Na_2_SO_4_ was added. The tube was vortexed again at 2500 rpm for 10 min. Then it was centrifuged for 10 min (3500 × *g*). The supernatant was placed in a 50 mL centrifuge tube and supplemented with ethyl acetate (10 mL) for re-extraction. Two supernatants were collected, merged, and concentrated in water bath at 40 ℃. The supernatants were supplemented with 2.5 mL acetonitrile to redissolve before filter-press cleanup procedure.

### 2.4 Operation procedures of filter-press cleanup column

The workflow of filter-press cleanup column is presented in Fig. 1, and in the demonstrating video (See supplementary materials).

(i) Five 5 mL centrifuge tubes were placed in a tube rack.
(ii) 2.5 mL of extract was added to each centrifuge tube.
(iii) Each centrifuge tube was placed with a 3 mL filter-press cleanup column (Fig. 1).
(iv) The filter-press cleanup column was pushed manually to the bottom of the centrifuge tube using a flat plate. The extract was passed through the r-DSPE sorbent layer under pressure.
(v) Purified extract was collected and ready for analysis.

### 2.5 UHPLC-HRMS instrumentation and conditions

Data acquisition was achieved using an UHPLC-Q-Orbitrap-HRMS instrument (Thermo Fisher Scientific, Massachusetts, USA). The analyses were conducted on a UHPLC (Dionex Ultimate 3000, Thermofisher) coupled with an Orbitrap HRMS instrument (Q-Exactive, Thermofisher). Chromatographic analysis was performed using an Agilent Eclipse Plus C_18_ RRHD (150 mm × 3.0 mm, 1.8 μm particle size) column. The column temperature was 40 °C. Mobile phases were (A) water with 5 mM ammonium acetate containing formic acid (0.2%, V/V) and (B) methanol containing formic acid (0.2%, V/V). The injection volume was 4.0 μL. The mobile phases’ flow rate was 400 µL min^−1^. After separation, the analytes were imported into the Orbitrap HRMS for identification and quantification. The analytes were ionized using a heated electrospray interface. The UHPLC gradient elution program for target analytes except tetracycline antibiotics was shown in Table S1. The UHPLC gradient elution program for tetracycline antibiotics was presented in Table S2. The MS conditions for each analyte were presented in Table S3. Ion source parameters were: 4.0 kV Spray voltage for the positive mode; 400 °C Aux gas heater temperature; 350 °C Capillary temperature; 50.0 S-lens RF level; 50 arbitrary unit (Arb) Sheath gas (N_2_) pressure, 1 Arb Sweep gas (N_2_) pressure and 15 Arb Auxiliary gas (N_2_).

Both full scan MS and data-dependent MS^2^ (dd-MS^2^) mode were used for analysis. Parameters for the full scan MS were: 70′000 Orbitrap resolution; *m*/*z* 70–1050 mass range; 60 % RF lens; 100 ms maximum injection time (IT); 3e^6^ normalized automatic gain control (AGC). When the target compound mass was detected in full scan MS mode, the target MS^2^ scan mode would be triggered.

The dd-MS^2^ mode parameters were: 17′500 Orbitrap resolution; 1.5 *m*/*z* isolation window; 1e^5^ AGC; 50 ms maximum IT.

### 2.6 HRMS database

For quick screening of target analytes, an HRMS database of 112 target veterinary drugs was established. Data-dependent MS^2^ and full scan MS modes were used to identify standard solutions of each drug. The parameters containing precursor, retention time and MS^2^ fragment ions, ionic ratio, as well as mass accuracy tolerance were used to identify and confirm target veterinary drugs.

### 2.6 Method validation

Data analysis of the developed method were performed using TraceFinder TM 5.1 software (Thermo Fisher Scientific). Based on the European Commission Regulation 2021/808, parameters of the developed method were verified including linearity, sensitivity, recovery, precision, as well as matrix effects (MEs). Series standard solutions ranging from 1.0 to 100.0 μg·kg^−1^ were detected to construct matrix-matched calibration curves of each analyte. Method sensitivity was investigated by calculating limit of detection (LOD) and limit of quantification (LOQ). Method precision was presented by recovery. Method accuracy was expressed by relative standard deviation (RSD). Afterwards, fish samples spiked with standard solutions at three concentrations was used to investigate recovery. Each spiked concentration was done with 6 replicates in parallel on the same day to calculate recovery and intra-day RSD. Inter-day RSDs were conducted at three doses on three consecutive days.

The calculation formula of MEs was as follows:

MEs= (Area_m_/Area_s_-1)*100%.

Where Area_s_ is the standard solution’s peak area, Area_m_ is the matrix-matched solution’s peak area with the same concentration as Area_s_.

## 3 Results and discussion

### 3.1 Uhplc-q-orbitrap HRMS instrumentation and conditions

UHPLC-Q-Orbitrap HRMS has both high quantitative detection abilities of HRMS and high chromatographic separation abilities of UHPLC. Rapid screening and identification of targeted analytes in fish samples was performed according to precursor, MS^2^ fragment ions, with a mass accuracy tolerance, and their retention time. Firstly, high precision precusor ion peaks of 112 target compounds were obtained by HRMS in full scan mode using sequential positive and negative ionization. Optimum ionization mode of each analyte was selected by comparing their response under positive and negative ionization modes, respectively. All the precursor ions of 112 analytes tended to form adducts of [M + H]^+^. The measured mass errors of 112 target compounds were all less than 5 ppm compared to their theoretical values (Table S3). Further, the fragment ions of each compound were obtained in the dd MS^2^ mode. Top two abundant fragment ions of each compound were used as qualitative ions (Table S2).

**Fig. 2:**
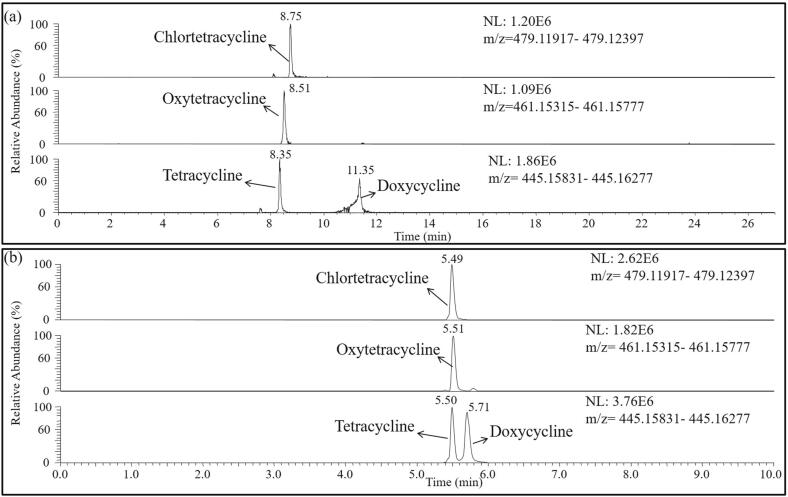
Extracted ion chromatograms of tetracycline antibiotics standard solution (32 μg·L^-1^) under different gradient elution programs (a) and (b).

The separation conditions of HPLC are crucial to the separation effect of target compounds, which determines the stability of retention time of each target compound, and then affects the accuracy of qualitative results of HRMS. 112 target compounds were all retained on the reverse-phase column. The separation performance of 112 target compounds was studied using 5 mM of ammonium acetate containing formic acid (0.2%, V/V) and methanol containing formic acid (0.2%, V/V) as mobile phase. The experimental results showed that 108 compounds except tetracycline antibiotics could be well separated within 27 min using 5 mM of ammonium acetate containing formic acid (0.2%, V/V), methanol containing formic acid (0.2%, V/V) as mobile phase and using the gradient elution procedure in Table S1. As shown in Fig. 2(a), the chromatogram peaks of 4 tetracycline antibiotics were poor. To improve the accuracy and reliability of test results, tetracycline antibiotics were analyzed separately under another liquid chromatography condition. Through detailed investigation, the effective separation of tetracycline antibiotics was achieved by adjusting the flow rate and gradient conditions of mobile phase without changing the chromatographic column (Fig. 2 (b)). The optimum UHPLC gradient elution program for tetracycline antibiotics was shown in Table S2. The retention times of 112 compounds were also shown in Table S3.

### 3.2 Sample preparation

#### 3.2.1 Development of the extraction method

The extraction step is a key step in analysis of veterinary medicines in aquatic products. The 112 target drugs cover different categories, including beta-agonists, macrolides, sulfonamides, quinolones, tetracyclines, benzimidazoles, nitroimidazoles, tranquilizers, malachite green and crystal violet. The polarity of target drugs differs significantly. Acetonitrile (Gadaj et al., 2014) and ethyl acetate (Ke et al., 2014) are frequently used solvents for extraction of veterinary drugs in fish. When ethyl acetate is used as extract solvent, it is difficult to remove the lipids, which greatly impacts the recovery rate. Using acetonitrile as extract solvent can avoid high fraction extraction of lipids from sample, and leads to good protein precipitation effect. However, it is not ideal for extracting some compounds with weak polarity.

In order to maximumly extract the compounds with different properties. a two-step extraction method was adopted according to our previous work with some modifications (Kong et al., 2018). First, acetonitrile was used for extraction. Then, 5 g of anhydrous Na_2_SO_4_ was added to the extraction solvent to remove water-soluble impurities. Afterwards, ethyl acetate was used for secondary extraction.

#### 3.2.2 Development of the cleanup procedure

##### 3.2.2.1 Cleanup sorbents

**Fig. 3:**
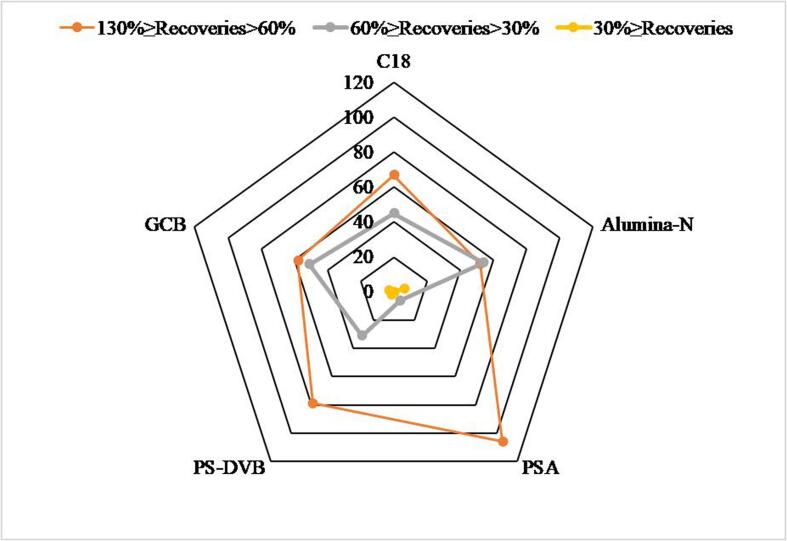
Number of detected target analytes after cleanup with different r-DSPE sorbents on the matrix-matched solution (16.0 μg·kg^−1^, n = 3).

The cleanup step is critical in the analysis of veterinary medicines in aquatic products. The matrix of aquatic products is complex and contains many interfering components, such as lipids. In the process of extraction, these interfering components can enter the extract and affect the accuracy of the detection results. Here we presented a filter-press cleanup column that allows rapid single-step cleanup operation in sample preparation. Different r-DSPE sorbent was packed into filter-press type cleanup column for evaluation, such as PSA, C_18_, PS-DVB, GCB, and alumina-N. The amount of PSA, C_18_, PS-DVB, GCB, and alumina-N were all 100 mg. As displayed in Fig. 3, most of target analytes were recovered at the satisfactory range (from 60% to 130%) when PSA was selected as sorbent to absorb interfering compounds in the matrix. As an anion exchange sorbent, the amino group in the PSA sorbent can be protonated in the acidic extract solution and forms electrical interaction with carbonyl groups in the interacting substances. This leads to high absorption of interacting acid compounds which are the main interfering substances in the matrix. Therefore, PSA was selected as r-DSPE sorbent for purification.

##### 3.2.2.2 The amounts of cleanup sorbents

**Fig. 4:**
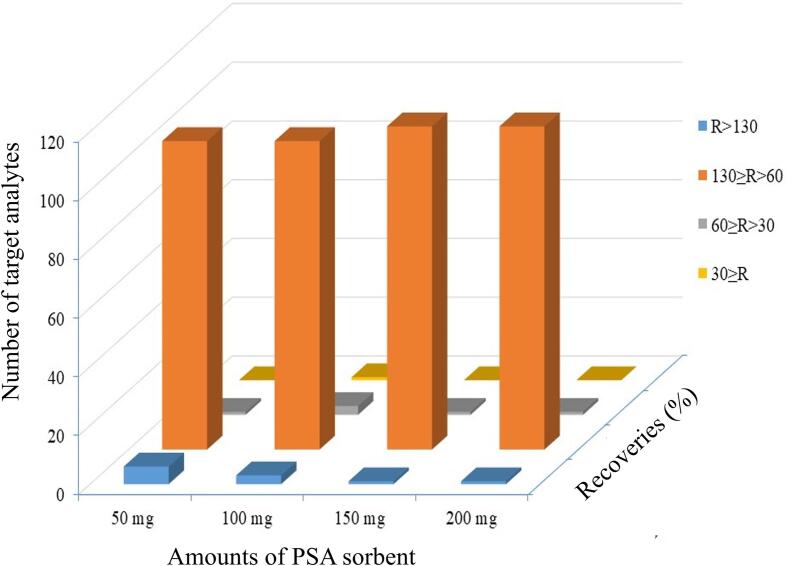
Number of detected target analytes after cleanup with different amounts of PSA on the matrix-matched solution (16.0 μg·kg^−1^, n = 3).

Fish samples are complex and contain a certain amount of proteins, fats and phospholipids, which can significantly influence the filter-press cleanup efficiency of the extract solution. The amount of cleanup sorbent can have a significant impact on the efficiency of removing interfering compounds from extract solution. The effects of the cleanup of serials amounts of PSA sorbent were assessed. As shown in Fig. 4, when the amount of PSA sorbents was 150 mg, most analytes were recovered at the satisfactory range (from 60% to 130%). To ensure the purification effect of proteins, fats and phospholipids in fish matrix, 150 mg PSA was chosen as the optimum cleanup amount.

#### 3.2.3 Evaluation of MEs

**Fig. 5:**
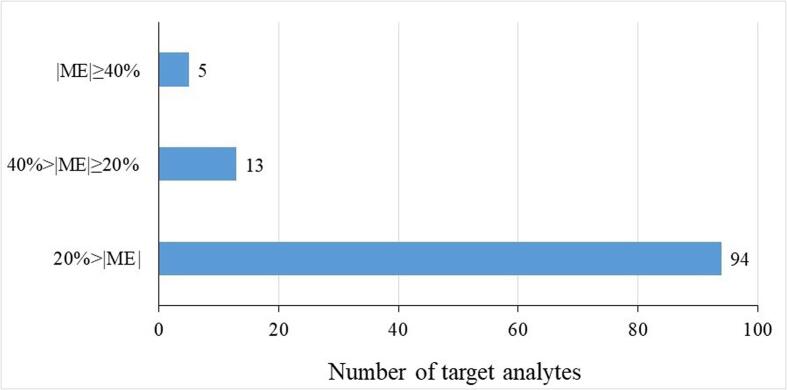
ME values of 112 analytes spiked in the extract solution of grass carp muscle (16.0 μg kg^−1^, n = 4).

The ME is a generic phenomenon that can enhance or suppress signals of target analytes due to influence of interfering substances on the ionization process. In our study, MEs were evaluated and calculated. As shown in Fig. 5 and Table S4, MEs varies from −68.2 to 56.9%. It was noted that the absolute ME values of 5 drugs were higher than 40 % and 13 drugs were found with absolute ME values in the range of 20–40%. All of the other analytes were observed with weak ME (less than 20%). To obtain an accurate result for quantification, matrix-matched calibration method was adopted to compensate for the impact of MEs on the test results.

### 3.3 Method validation

After optimizing the analysis parameters, the method validation results were summarized in Table S4 and Table S5. Matrix-matched calibration curves of target compounds were established from 1.0 to 100.0 μg·kg^−1^, as shown in Table S4. The coefficient of curves was all higher than 0.94. LODs and LOQs were assessed considering S/N of >3 and >10, respectively. LODs and LOQs for these compounds were 0.5 and 2.0 μg·kg^−1^, respectively (Table S4).

Furthermore, blank grass carp spiked at three concentrations (2.0, 5.0, 16.0 μg·kg^−1^) were prepared to validate recoveries and RSDs. As a result, recoveries of 35.3 to 138.4 % were obtained (Table S5). Intra-day RSDs were found to range from 1.9 to 26.1%. Inter-day RSDs were also evaluated and found to range from 1.8% to 31.8%. These results confirm that the developed method is reliable enough for the monitoring of drug residues in aquatic products.

### 3.4 Comparisons of single-step cleanup approaches

To demonstrate the practicability and the advantages of this method, a comparison of diverse single-step cleanup methods for rapid screening of drugs in fish samples by HRMS is summarized in Table S6. Compared to the m-PFC cleanup approach, the developed method is capable of batch purification, and up to five samples can be manually processed in parallel. Sin-QuEChERS column and these methods are both filter-press single-step cleanup methods. However, the advantages of our developed filter-press single-step cleanup method are obvious. It allows all extracts in the centrifuge tube to pass through the r-DSPE layer. Futhermore, the purification process can be achieved manually with five or more samples in parallel. Compared with the Sin-QuEChERS column, the developed novel filter-press single-step cleanup approach is more cost-efficient and is also easier to operate.

### 3.5 Application in real samples

**Fig. 6:**
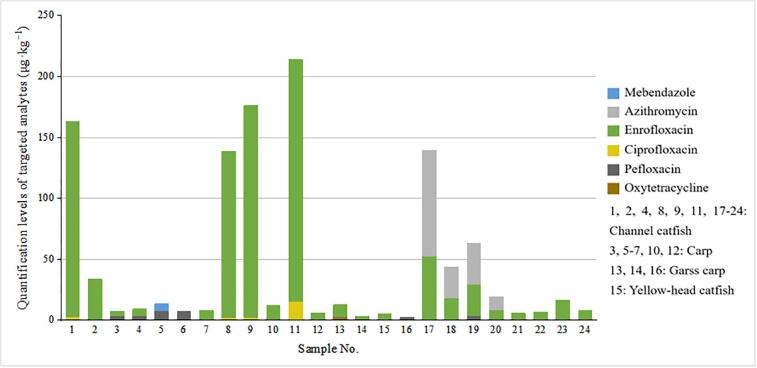
Detected concentrations of drug residues in real samples.

The presented approaches were successfully used for rapid screening and quantification of the studied analytes in 28 live fish samples obtained randomly from freshwater aquaculture farms (Table S7). The results are shown in Fig. 6 and Table S7. Oxytetracycline, pefloxacin, ciprofloxacin, enrofloxacin, azithromycin, and mebendazole were found and quantified in real samples. The residue level of enrofloxacin was highest, and the highest detected concentration was 198.119 μg·kg^−1^, which exceed the maximum residue limit MRL of China’s regulation (GB 31650–2019, 100 μg·kg^−1^). Its metabolite, ciprofloxacin, was also detected in four samples with concentrations ranging from 2.2 to 15.6 μg·kg^−1^. Pefloxacin was found in five samples with detected concentrations ranging from 2.6 − 7.9 μg·kg^−1^. These tested samples were also unqualified according to the regulations set in GB 31650–2019. The result further demonstrates the applicability and efficiency of this method in monitoring of veterinary drug residues in aquatic products.

## 4 Conclusion

This study proposed a new filter-press single-step cleanup method and utilized the established HPLC-Q-Orbitrap HRMS method for the rapid screening and quantification of 112 veterinary drugs in fish samples. The results showed that 150 mg of PSA exhibited the best purification effect. The spiked recovery rates ranged from 35.3% to 138.4%, with RSDs ranging from 1.8% to 31.8%. 84% of the analytes were detected with matrix effects below 20%.

To our knowledge, this was the first report of filter-press cleanup column and its use for multiple samples being manually parallel purified in a single step. The developed filter-press single-step cleanup method was simple and easy to operate, and it could also be used for analyzing other drug residues in aquatic products. This method exhibited good advantages and potential as a routine sample preparation method, providing a reliable approach for the automation of sample purification processes.

## CRediT authorship contribution statement

**Jincheng Li:** Conceptualization, Funding acquisition, Resources, Supervision, Writing – review & editing. **Ruidong Zhou:** Methodology, Formal analysis. **Guangxin Yang:** Formal analysis, Visualization. **Essy Kouadio Fodjo:** Writing – review & editing. **Tengwang Feng:** Formal analysis. **Huiwu Sun:** Resources, Supervision. **Dongmei Huang:** Resources, Supervision. **Cong Kong:** Conceptualization, Writing – review & editing. **Huan Liu:** Resources, Supervision, Writing – review & editing.

## Declaration of Competing Interest

The authors declare that they have no known competing financial interests or personal relationships that could have appeared to influence the work reported in this paper.

## Data Availability

Data will be made available on request.
