# Supplementary material for: Novel filter-press single-step cleanup approach facilitated rapid screening and accurate quantification of 112 veterinary drugs in aquatic products
Source: Food Chem X. 2023 Aug 19;19:100846. doi: 10.1016/j.fochx.2023.100846 (PMC10534240; doi:10.1016/j.fochx.2023.100846)
Supplement: Supplementary data 2 [file mmc2.docx]

Table S1 The UHPLC gradient elution program for target analytes except tetracycline antibiotics

| Time (min) | Flow rate  (mL min^-1^) | A ( water(Containing 5 mmol·L^-1^ ammonium acetate ) with 0.2% formic acid)（%, *v/v*） | B (methanol with 0.2% formic acid)（%, *v/v*） |
| --- | --- | --- | --- |
| 0.0 | 0.40 | 95.0 | 5.0 |
| 1.0 | 0.40 | 95.0 | 5.0 |
| 3.0 | 0.40 | 80.0 | 20.0 |
| 8.0 | 0.40 | 50.0 | 50.0 |
| 19.0 | 0.40 | 5.0 | 95.0 |
| 23.0 | 0.40 | 5.0 | 95.0 |
| 23.1 | 0.40 | 95.0 | 5.0 |
| 27.0 | 0.40 | 95.0 | 5.0 |

Table S2 The UHPLC gradient elution program for tetracycline antibiotics

| Time (min) | Flow rate  (mL·min^-1^) | A ( water(containing 5 mmol·L^-1^ Ammonium acetate ) with 0.2% formic acid)（%, *v/v*） | B (methanol with 0.2% formic acid)（%, *v/v*） |
| --- | --- | --- | --- |
| 0.0 | 0.50 | 99.0 | 1.0 |
| 1.5 | 0.50 | 99.0 | 1.0 |
| 3.0 | 0.50 | 80.0 | 20.0 |
| 4.0 | 0.50 | 0.0 | 100.0 |
| 8.5 | 0.50 | 0.0 | 100.0 |
| 8.65 | 0.50 | 99.0 | 1.0 |
| 10.0 | 0.50 | 99.0 | 1.0 |

Table S3 Information of 112 veterinary drugs and their MS conditions in UPLC-Q-Orbitrap-HRMS

| No. | Target compound | Retention time (min) | Molecule formula | CAS No. | Mass to charge ratio (m/z) | | | | Product ions (m/z) |
| --- | --- | --- | --- | --- | --- | --- | --- | --- | --- |
|  |  |  |  |  | Adduct | Theoretical | Measured | Error (ppm) |  |
| 1 | Tetracycline | 5.46 | C_22_H_24_N_2_O_8_ | 60-54-8 | [M+H]^+^ | 445.16054 | 445.16000 | -1.21 | 410.12323,  154.04987 |
| 2 | Chlortetracycline | 5.58 | C_22_H_23_ClN_2_O_8_ | 57-62-5 | [M+H]^+^ | 479.12157 | 479.12387 | 4.80 | 444.08444, 462.09518 |
| 3 | Oxytetracycline | 5.48 | C_22_H_24_N_2_O_9_ | 79-57-2 | [M+H]^+^ | 461.15546 | 461.15494 | -1.13 | 426.11823, 201.05473 |
| 4 | Doxycycline | 5.70 | C_22_H_24_N_2_O_8_ | 564-25-0 | [M+H]^+^ | 445.16054 | 445.16049 | -0.11 | 428.13446  321.07575 |
| 5 | Trimethoprim | 7.53 | C_14_H_18_N_4_O_3_ | 738-70-5 | [M+H]^+^ | 291.14517 | 291.14490 | -0.93 | 261.09818,  230.11626 |
| 6 | Sulfaphenazole | 9.95 | C_15_H_14_N_4_O_2_S | 526-08-9 | [M+H]^+^ | 315.09102 | 315.09061 | -1.30 | 158.07137,  160.08696 |
| 7 | Sulfabemzamide | 9.59 | C_13_H_12_N_2_O_3_S | 127-71-9 | [M+H]^+^ | 277.06414 | 277.06400 | -0.51 | 156.01141,  108.04481 |
| 8 | Sulfapyridine | 6.81 | C_11_H_11_N_3_O_2_S | 144-83-2 | [M+H]^+^ | 250.06447 | 250.06419 | -1.12 | 156.01147,  108.04485 |
| 9 | Sulfameter | 8.19 | C_11_H_12_N_4_O_3_S | 651-06-9 | [M+H]^+^ | 281.07029 | 281.06995 | -1.21 | 156.01143,  126.06643 |
| 10 | Sulfamoxol | 7.69 | C_11_H_13_N_3_O_3_S | 729-99-7 | [M+H]^+^ | 268.07504 | 268.07474 | -1.12 | 156.0114,  113.0713 |
| 11 | Sulfamethazine | 8.06 | C_12_H_14_N_4_O_2_S | 57-68-1 | [M+H]^+^ | 279.09102 | 279.09076 | -0.93 | 156.01138,  124.08692 |
| 12 | Sulfisoxazole | 9.05 | C_11_H_13_N_3_O_3_S | 127-69-5 | [M+H]^+^ | 268.07504 | 268.07474 | -1.12 | 156.01144,  113.0713 |
| 13 | Sulfisomidine | 6.24 | C_12_H_14_N_4_O_2_S | 515-64-0 | [M+H]^+^ | 279.09102 | 279.0907 | -1.15 | 156.01138,  124.08692 |
| 14 | Sulfamethoxazole | 8.67 | C_10_H_11_N_3_O_3_S | 723-46-6 | [M+H]^+^ | 254.05939 | 254.05913 | -1.02 | 156.0114,  108.04482 |
| 15 | Sulfamerazine | 7.16 | C_11_H_12_N_4_O_2_S | 127-79-7 | [M+H]^+^ | 265.07537 | 265.07507 | -1.13 | 156.01141,  110.07165 |
| 16 | Sulfamethizole | 7.80 | C9H10N4O2S2 | 144-82-1 | [M+H]^+^ | 271.03179 | 271.03146 | -1.22 | 156.01138,  108.04482 |
| 17 | Sulfamehoxypyridazine | 7.71 | C_11_H_12_N_4_O_3_S | 80-35-3 | [M+H]^+^ | 281.07029 | 281.07004 | -0.89 | 156.01141,  126.06641 |
| 18 | Sulfadimethoxine | 10.45 | C_12_H_14_N_4_O_4_S | 155-91-5 | [M+H]^+^ | 311.08085 | 311.08047 | -1.22 | 156.07684,  108.04478 |
| 19 | Sulfamonomethoxine | 8.86 | C_11_H_12_N_4_O_3_S | 1220-83-3 | [M+H]^+^ | 281.07029 | 281.07001 | -1.00 | 156.01143,  108.04481 |
| 20 | Sulfaquinoxalin | 10.79 | C_14_H_12_N_4_O_2_S | 59-40-5 | [M+H]^+^ | 301.07537 | 301.07510 | -0.90 | 156.01144,  108.04483 |
| 21 | Sulfadoxine | 9.12 | C_12_H_14_N_4_O_4_S | 2447-57-6 | [M+H]^+^ | 311.08085 | 311.08041 | -1.41 | 156.01141,  108.04482 |
| 22 | Sulfaclozone | 10.18 | C_10_H_9_ClN_4_O_2_S | 102-65-8 | [M+H]^+^ | 285.02075 | 285.02075 | 0.00 | 156.0114,  130.01685 |
| 23 | Sulfachloropyridazine | 8.59 | C_10_H_9_ClN_4_O_2_S | 80-32-0 | [M+H]^+^ | 285.02075 | 285.02048 | -0.95 | 156.0114,  108.04482 |
| 24 | Sulfadiazine | 6.14 | C_10_H_10_N_4_O_2_S | 68-35-9 | [M+H]^+^ | 251.05972 | 251.05956 | -0.64 | 156.01141,  108.04483 |
| 25 | Sulfatroxazole | 8.78 | C_11_H_13_N_3_O_3_S | 23256-23-7 | [M+H]^+^ | 268.07504 | 268.07474 | -1.12 | 156.01147,  108.04487 |
| 26 | Sulfathiazole | 6.47 | C_9_H_9_N_3_O_2_S_2_ | 72-14-0 | [M+H]^+^ | 256.02089 | 256.02063 | -1.02 | 156.0114,  108.04482 |
| 27 | Sulfaethoxypyridazine | 9.80 | C_12_H_14_N_4_O_3_S | 963-14-4 | [M+H]^+^ | 295.08594 | 295.08569 | -0.85 | 156.0114,  140.08191 |
| 28 | Sulfapyrazole | 10.67 | C_16_H_16_N_4_O_2_S | 852-19-7 | [M+H]^+^ | 329.10667 | 329.10638 | -0.88 | 172.08694,  156.01147 |
| 29 | Fleroxacin | 7.67 | C_17_H_18_F_3_N_3_O_3_ | 79660-72-3 | [M+H]^+^ | 370.1373 | 370.1369 | -1.08 | 326.14777,  269.08981 |
| 30 | Sparfloxacin | 9.71 | C_19_H_22_F_2_N_4_O_3_ | 111542-93-9 | [M+H]^+^ | 393.17327 | 393.17297 | -0.76 | 349.18372,  292.12582 |
| 31 | Gatifloxacin | 9.29 | C_19_H_22_FN_3_O_4_ | 112811-59-3 | [M+H]^+^ | 376.16671 | 376.16641 | -0.80 | 332.17712,  261.10373 |
| 32 | Lomefloxacin | 8.65 | C_17_H_19_F_2_N_3_O_3_ | 98079-51-7 | [M+H]^+^ | 352.14672 | 352.14639 | -0.94 | 308.15707,  265.11481 |
| 33 | Pefloxacin | 7.98 | C_17_H_20_FN_3_O_3_ | 70458-92-3 | [M+H]^+^ | 334.15615 | 334.15585 | -0.90 | 290.16653,  233.10878 |
| 34 | Ofloxacin | 7.94 | C_18H20_FN_3_O_4_ | 82419-36-1 | [M+H]^+^ | 362.15106 | 362.15079 | -0.75 | 318.1615,  261.10361 |
| 35 | Norfloxacin | 8.16 | C_16_H_18_FN_3_O_3_ | 70458-96-7 | [M+H]^+^ | 320.1405 | 320.14011 | -1.22 | 276.15064,  233.10876 |
| 36 | Danpfloxacin | 8.49 | C_19_H_20_FN_3_O_3_ | 112398-08-0 | [M+H]^+^ | 358.15615 | 358.15588 | -0.75 | 255.05643,  82.06593 |
| 37 | Difloxacin | 8.82 | C_21_H_19_F_2_N_3_O_3_ | 98106-17-3 | [M+H]^+^ | 400.14672 | 400.14648 | -0.60 | 356.15717,  299.09927 |
| 38 | Enrofloxacin | 8.46 | C_19_H_22_FN_3_O_3_ | 93106-60-6 | [M+H]^+^ | 360.1718 | 360.17108 | -2.00 | 316.18222,  245.10869 |
| 39 | Flumequine | 13.01 | C_14_H_12_FNO_3_ | 42835-25-6 | [M+H]^+^ | 262.0874 | 262.08719 | -0.80 | 238.05118,  220.0407 |
| 40 | Oxolinic acid | 11.00 | C_13_H_11_NO_5_ | 14698-29-4 | [M+H]^+^ | 262.071 | 262.07083 | -0.65 | 244.06046,  234.03989 |
| 41 | Sarafloxacin | 9.03 | C_20_H_17_F_2_N_3_O_3_ | 98105-99-8 | [M+H]^+^ | 386.13107 | 386.13077 | -0.78 | 342.14157,  299.09933 |
| 42 | Orbifloxacin | 8.76 | C_19_H_20_F_3_N_3_O_3_ | 113617-63-3 | [M+H]^+^ | 396.15295 | 396.15271 | -0.61 | 352.16339,  295.10556 |
| 43 | Pipemidic acid | 7.34 | C_14_H_17_N_5_O_3_ | 51940-44-4 | [M+H]^+^ | 304.14042 | 304.13989 | -1.74 | 217.10867,  189.0773 |
| 44 | Ciprofloxacin | 8.36 | C_17_H_18_FN_3_O_3_ | 85721-33-1 | [M+H]^+^ | 332.1405 | 332.14014 | -1.08 | 288.15097,  231.05675 |
| 45 | Marbofloxacin | 7.53 | C_17_H_19_FN_4_O_4_ | 115550-35-1 | [M+H]^+^ | 363.14631 | 363.14594 | -1.02 | 363.14636,  72.08163 |
| 46 | Nalidixic acid | 12.64 | C_12_H_12_N_2_O_3_ | 389-08-2 | [M+H]^+^ | 233.09207 | 233.09187 | -0.86 | 205.06104,  187.05043 |
| 47 | Cinoxacin | 10.48 | C_12_H_10_N_2_O_5_ | 28657-80-9 | [M+H]^+^ | 263.06625 | 263.06601 | -0.91 | 217.06105,  189.02971 |
| 48 | Enoxacin | 8.00 | C_15_H_17_FN_4_O_3_ | 74011-58-8 | [M+H]^+^ | 321.13575 | 321.13553 | -0.69 | 303.12607,  234.10452 |
| 49 | Malachite green | 14.08 | C_23_H_24_N_2_ | 2437-29-8 | [M+H]^+^ | 329.20123 | 329.20111 | -0.36 | 313.16995,  208.11224 |
| 50 | Crystal violet | 15.72 | C_25_H_29_N_3_ | 548-62-9 | [M+H]^+^ | 372.24342 | 372.24298 | -1.18 | 356.21255，  251.15428 |
| 51 | Diazepam | 16.06 | C_16_H_13_ClN_2_O | 439-14-5 | [M+H]^+^ | 285.07892 | 285.07858 | -1.19 | 193.08881,  154.04192 |
| 52 | Triazolam | 14.46 | C_17_H_12_Cl_2_N_4_ | 28911-01-5 | [M+H]^+^ | 343.05118 | 343.05081 | -1.08 | 315.03259,  308.08246 |
| 53 | Lorazepam | 14.50 | C_15_H_10_Cl_2_N_2_O_2_ | 846-49-1 | [M+H]^+^ | 321.01921 | 321.01859 | -1.93 | 303.00867,  275.01361 |
| 54 | Nordiazepam | 15.46 | C_15_H_11_ClN_2_O | 1088-11-5 | [M+H]^+^ | 271.06327 | 271.06305 | -0.81 | 165.02155,  140.02623 |
| 55 | Oxazepam | 14.56 | C_15_H_11_ClN_2_O_2_ | 604-75-1 | [M+H]^+^ | 287.05818 | 287.05798 | -0.70 | 269.04749,  241.05258 |
| 56 | Methaqualone | 14.16 | C_16_H_14_N_2_O | 72-44-6 | [M+H]^+^ | 251.11789 | 251.11769 | -0.80 | 132.08099,  91.05423 |
| 57 | Promethazine | 12.80 | C_17_H_20_N_2_S | 60-87-7 | [M+H]^+^ | 285.142 | 285.14175 | -0.88 | 198.03731,  86.09708 |
| 58 | Temazepam | 15.00 | C_16_H_13_ClN_2_O_2_ | 846-50-4 | [M+H]^+^ | 301.07383 | 301.07333 | -1.66 | 283.0632,  255.06822 |
| 59 | Chlorpromazine | 14.60 | C_17_H_19_ClN_2_S | 50-53-3 | [M+H]^+^ | 319.10302 | 319.10269 | -1.03 | 86.0971,  246.01387 |
| 60 | Nitrazepam | 13.51 | C_15_H_11_N_3_O_3_ | 146-22-5 | [M+H]^+^ | 282.08732 | 282.08704 | -0.99 | 268.08429,  236.09445 |
| 61 | Estazolam | 14.10 | C_16_H_11_ClN_4_ | 29975-16-4 | [M+H]^+^ | 295.0745 | 295.0744 | -0.34 | 267.05579,  205.07608 |
| 62 | Alprazolam | 14.60 | C_17_H_13_ClN_4_ | 28981-97-7 | [M+H]^+^ | 309.09015 | 309.08978 | -1.20 | 281.07132,  205.07605 |
| 63 | Clenbuterol | 9.03 | C_12_H_18_Cl_2_N_2_O | 37148-27-9 | [M+H]^+^ | 277.0869 | 277.08658 | -1.15 | 203.0137,  132.06833 |
| 64 | Cimaterol | 5.42 | C_12_H_17_N_3_O | 54239-37-1 | [M+H]^+^ | 220.14444 | 220.1442 | -1.09 | 160.0871,  202.13399 |
| 65 | Bamethan | 6.63 | C_12_H_19_NO_2_ | 3703-79-5 | [M+H]^+^ | 210.14886 | 210.14873 | -0.62 | 192.13831,  136.07581 |
| 66 | Bambuterol | 10.17 | C_18_H_29_N_3_O_5_ | 81732-65-2 | [M+H]^+^ | 368.218 | 368.21771 | -0.79 | 294.14478,  312.1556 |
| 67 | Phenylethanolamine A | 11.44 | C_19_H_24_N_2_O_4_ | 1346746-81-3 | [M+H]^+^ | 345.18088 | 345.18066 | -0.64 | 327.17029,  150.09145 |
| 68 | Ractopamine | 8.24 | C_18_H_23_NO_3_ | 97825-25-7 | [M+H]^+^ | 302.17507 | 302.17468 | -1.29 | 121.0653,  107.04954 |
| 69 | Ritodrine | 6.91 | C_17_H_21_NO_3_ | 26652-09-5 | [M+H]^+^ | 288.15942 | 288.15903 | -1.35 | 270.14874,  121.06505 |
| 70 | Mabuterol | 9.93 | C_13_H_18_ClF_3_N_2_O | 56341-08-3 | [M+H]^+^ | 311.11325 | 311.11285 | -1.29 | 237.03993,  217.03386 |
| 71 | Mapenterol | 10.83 | C_14_H_20_ClF_3_N_2_O | 95656-68-1 | [M+H]^+^ | 325.1289 | 325.12869 | -0.65 | 237.04002,  217.03389 |
| 72 | Penbutolol | 14.65 | C_18_H_29_NO_2_ | 36507-48-9/38363-40-5 | [M+H]^+^ | 292.22711 | 292.22622 | -3.05 | 236.16455,  133.06499 |
| 73 | Salmeterol | 14.63 | C_25_H_37_NO_4_ | 89365-50-4 | [M+H]^+^ | 416.27954 | 416.27914 | -0.96 | 398.2694,  380.25851 |
| 74 | Terbutaline | 5.62 | C_12_H_19_NO_3_ | 23031-25-6 | [M+H]^+^ | 226.14377 | 226.14348 | -1.28 | 152.0706,  125.05987 |
| 75 | Tulobuterol | 9.74 | C_12_H_18_ClNO | 41570-61-0 | [M+H]^+^ | 228.11497 | 228.11475 | -0.96 | 154.04181,  118.0654 |
| 76 | Cimbuterol | 6.34 | C_13_H_19_N_3_O | 54239-39-3 | [M+H]^+^ | 234.16009 | 234.15984 | -1.07 | 160.08694,  143.06047 |
| 77 | Bromobuterol | 9.78 | C_12_H_18_Br_2_N_2_O | 41937-02-4 | [M+H]^+^ | 364.98586 | 364.98560 | -0.71 | 290.91336,  211.99498 |
| 78 | Isoxsuprine | 9.91 | C_18_H_23_NO_3_ | 395-28-8 | [M+H]^+^ | 302.17507 | 302.17477 | -0.99 | 284.1644,  107.04955 |
| 79 | Isoetarine | 5.74 | C_13_H_21_NO_3_ | 530-08-5 | [M+H]^+^ | 240.15942 | 240.15909 | -1.37 | 222.14891,  148.07574 |
| 80 | Clenproperol | 8.12 | C_11_H_16_Cl_2_N_2_O | 38339-11-6 | [M+H]^+^ | 263.07125 | 263.07095 | -1.14 | 245.06075,  132.06841 |
| 81 | Clencyclohexerol | 7.52 | C_14_H_20_Cl_2_N_2_O_2_ | 157877-79-7 | [M+H]^+^ | 319.09746 | 319.09714 | -1.00 | 301.08685,  203.01373 |
| 82 | Roxithromycin | 15.35 | C_41_H_76_N_2_O_15_ | 80214-83-1 | [M+H]^+^ | 837.53185 | 837.53210 | 0.30 | 679.43726,  158.11769 |
| 83 | Clarithromycin | 15.09 | C_38_H_69_NO_13_ | 81103-11-9 | [M+H]^+^ | 748.48417 | 748.48309 | -1.44 | 158.11772,  590.39658 |
| 84 | Azithromycin | 10.57 | C_38_H_72_N_2_O_12_ | 83905-01-5 | [M+H]^+^ | 749.5158 | 749.51477 | -1.37 | 158.11763,  116.1073 |
| 85 | Clindamycin | 11.69 | C_18_H_33_ClN_2_O_5_S | 18323-44-9 | [M+H]^+^ | 425.18715 | 425.18674 | -0.96 | 126.12789  377.18414 |
| 86 | Timicosin | 11.47 | C_46_H_80_N_2_O_13_ | 108050-54-0 | [M+H]^+^ | 869.57332 | 869.57367 | 0.40 | 696.46814,  174.11261 |
| 87 | Tylosin | 13.56 | C_46_H_77_NO_17_ | 1401-69-0 | [M+H]^+^ | 916.52643 | 916.52515 | -1.40 | 772.44775,  174.11261 |
| 88 | Rifaximin | 17.10 | C_43_H_51_N_3_O_11_ | 80621-31-4 | [M+H]^+^ | 786.35964 | 786.35895 | -0.88 | 754.33368,  151.07549 |
| 89 | Oleandomycin phosphate | 12.36 | C_35_H_61_NO_12_ | 7060-74-4 | [M+H]^+^ | 688.42665 | 688.42639 | -0.38 | 158.11765,  98.09699 |
| 90 | Albendazole | 14.42 | C_12_H_15_N_3_O_2_S | 54965-21-8 | [M+H]^+^ | 266.09577 | 266.09543 | -1.28 | 234.06972,  191.01497 |
| 91 | Albendazole-2-aminosulfone | 7.12 | C_10_H_13_N_3_O_2_S | 80983-34-2 | [M+H]^+^ | 240.08012 | 240.0798 | -1.33 | 165.05354,  133.06371 |
| 92 | Flubendazole | 14.39 | C_16_H_12_FN_3_O_3_ | 31430-15-6 | [M+H]^+^ | 314.09355 | 314.09323 | -1.02 | 282.06735,  123.02438 |
| 93 | Mebendazole | 13.84 | C_16_H_13_N_3_O_3_ | 31431-39-7 | [M+H]^+^ | 296.10297 | 296.10272 | -0.84 | 264.07693,  105.03407 |
| 94 | Hydroxymebendazole | 10.81 | C_16_H_15_N_3_OS | 60254-95-7 | [M+H]^+^ | 298.11862 | 298.11841 | -0.70 | 266.0924  298.11862 |
| 95 | Triclabendazole | 19.03 | C_14_H_9_Cl_3_N_2_OS | 68786-66-3 | [M+H]^+^ | 358.95739 | 358.95697 | -1.17 | 343.93423,  273.99634 |
| 96 | Oxibendazole | 11.91 | C_12_H_15_N_3_O_3_ | 20559-55-1 | [M+H]^+^ | 250.11862 | 250.11850 | -0.48 | 218.09264,  176.04568 |
| 97 | Thiabendazole | 8.46 | C_10_H_7_N_3_S | 148-79-8 | [M+H]^+^ | 202.04334 | 202.04314 | -0.99 | 175.0327  131.03067 |
| 98 | Cambendazol | 11.80 | C_14_H_14_N_4_O_2_S | 26097-80-3 | [M+H]^+^ | 303.09102 | 303.09076 | -0.86 | 261.04428,  217.05449 |
| 99 | Oxfendazole | 11.85 | C_15_H_13_N_3_O_3_S | 53716-50-0 | [M+H]^+^ | 316.07504 | 316.07489 | -0.47 | 159.04286,  191.03271 |
| 100 | Ciclobendazole | 11.74 | C_13_H_13_N_3_O_3_ | 31431-43-3 | [M+H]^+^ | 260.10297 | 260.10284 | -0.50 | 228.07687,  159.04277 |
| 101 | Febantel | 17.48 | C_20_H_22_N_4_O_6_S | 58306-30-2 | [M+H]^+^ | 447.13328 | 447.13300 | -0.63 | 383.08112,  280.05423 |
| 102 | Mebendazole-amine | 10.32 | C_14_H_11_N_3_O | 52329-60-9 | [M+H]^+^ | 238.09749 | 238.09723 | -1.09 | 105.0341,  95.04984 |
| 103 | Albendazole sulfoxide | 10.48 | C_12_H_15_N_3_O_3_S | 54029-12-8 | [M+H]^+^ | 282.09069 | 282.09042 | -0.96 | 208.08781,  191.06912 |
| 104 | Albendazole sulfone | 10.82 | C_12_H_15_N_3_O_4_S | 75184-71-3 | [M+H]^+^ | 298.0856 | 298.08545 | -0.50 | 266.05963,  224.01263 |
| 105 | Levamisole | 6.73 | C_11_H_12_N_2_S | 14769-73-4 | [M+H]^+^ | 205.0794 | 205.07936 | -0.20 | 178.06871,  123.02662 |
| 106 | Metronidazole | 6.07 | C_6_H_9_N_3_O_3_ | 443-48-1 | [M+H]^+^ | 172.07167 | 172.07149 | -1.05 | 128.04572,  140.04595 |
| 107 | Dimetridazole | 6.69 | C_5_H_7_N_3_O_2_ | 551-92-8 | [M+H]^+^ | 142.0611 | 142.06102 | -0.56 | 112.06352,  95.06102 |
| 108 | Ronidazole | 6.17 | C_6_H_8_N_4_O_4_ | 7681-76-7 | [M+H]^+^ | 201.06183 | 201.06166 | -0.85 | 140.04565,  55.04268 |
| 109 | Tinidazole | 7.20 | C_8_H_13_N_3_O4S | 19387-91-8 | [M+H]^+^ | 248.06995 | 248.06973 | -0.89 | 128.04579,  121.03214 |
| 110 | Ipronidazole | 10.19 | C_7_H_11_N_3_O_2_ | 14885-29-1 | [M+H]^+^ | 170.0924 | 170.09232 | -0.47 | 123.0926,  109.07654 |
| 111 | Hydroxyipronidazole | 9.06 | C_7_H_11_N_3_O_3_ | 35175-14-5 | [M+H]^+^ | 186.08732 | 186.08714 | -0.97 | 168.07693,  121.0764 |
| 112 | Secnidazole | 7.79 | C_7_H_11_N_3_O_3_ | 3366-95-8 | [M+H]^+^ | 186.08732 | 186.08723 | -0.48 | 128.04575,  59.0514 |

Table S4 The linearity, sensitivity, and MEs of the developed method

| Target Compounds | Linear equations | Coefficient of determination （R^2^） | Linear ranges (μg·kg ^-1^) | LODs  (μg·kg^-1^) | LOQs  (μg·kg^-1^) | MEs(%) |
| --- | --- | --- | --- | --- | --- | --- |
| Tetracycline | y =1.19e^5^x | 0.9781 | 1.0-100.0 | 0.5 | 2.0 | 11.7 |
| Chlortetracycline | y = 1.06e^5^+4.05e^4^x | 0.9824 | 1.0-100.0 | 0.5 | 2.0 | 26.8 |
| Oxytetracycline | y = 4.61e^5^+7.98e^4^x | 0.9925 | 1.0-100.0 | 0.5 | 2.0 | -31.6 |
| Doxycycline | y = 5.04e^5^+3.88e^5^x | 0.9902 | 1.0-100.0 | 0.5 | 2.0 | -38.8 |
| Trimethoprim | y = 3.07e^6^+4.95e^6^x | 0.9940 | 1.0-100.0 | 0.5 | 2.0 | 12.4 |
| Sulfaphenazole | y = -1.28e^5^+1.11e^6^x | 0.9965 | 1.0-100.0 | 0.5 | 2.0 | 23.3 |
| Sulfabemzamide | y = -1.02e^5^+4.02e^5^x | 0.9428 | 1.0-100.0 | 0.5 | 2.0 | 14.2 |
| Sulfapyridine | y = 2.88e^6^+1.86e^6^x | 0.9936 | 1.0-100.0 | 0.5 | 2.0 | 13.3 |
| Sulfameter | y = 9.86e^4^+1.32e^6^x | 0.9936 | 1.0-100.0 | 0.5 | 2.0 | 9.0 |
| Sulfamoxol | y = -9.75e^4^+1.26e^6^x | 0.9978 | 1.0-100.0 | 0.5 | 2.0 | -6.3 |
| Sulfamethazine | y = 2.69e^5^+1.98e^6^x | 0.9923 | 1.0-100.0 | 0.5 | 2.0 | 5.6 |
| Sulfisoxazole | y = -2.81e^2^+8.90e^5^x | 0.9937 | 1.0-100.0 | 0.5 | 2.0 | 10.8 |
| Sulfisomidine | y = 4.18e^5^+2.85e^6^x | 0.9921 | 1.0-100.0 | 0.5 | 2.0 | 30.2 |
| Sulfamethoxazole | y = 2.09e^5^+1.06e^6^*x | 0.9912 | 1.0-100.0 | 0.5 | 2.0 | 10.9 |
| Sulfamerazine | y = -9.23e^4^+1.42e^6^x | 0.9977 | 1.0-100.0 | 0.5 | 2.0 | 17.1 |
| Sulfamethizole | y = -5.91e^5^+5.23e^5^x | 0.9953 | 1.0-100.0 | 0.5 | 2.0 | 4.6 |
| Sulfamehoxypyridazine | y = 5.94e^4^+1.21e^6^x | 0.9983 | 1.0-100.0 | 0.5 | 2.0 | -4.5 |
| Sulfadimethoxine | y = 5.99e^3^+1.66e^6^x | 0.9964 | 1.0-100.0 | 0.5 | 2.0 | -0.8 |
| Sulfamonomethoxine | y = -2.80e^4^+6.63e^5^x | 0.9975 | 1.0-100.0 | 0.5 | 2.0 | 7.6 |
| Sulfaquinoxalin | y = -1.64e^5^+5.64e^5^x | 0.9987 | 1.0-100.0 | 0.5 | 2.0 | 6.4 |
| Sulfadoxine | y = 2.13e^5^+2.43e^6^x | 0.9966 | 1.0-100.0 | 0.5 | 2.0 | 9.8 |
| Sulfaclozone | y = -1.41e^5^+1.90e^5^x | 0.9932 | 1.0-100.0 | 0.5 | 2.0 | 6.5 |
| Sulfachloropyridazine | y = -6.86e^4^+3.83e^5^x | 0.9961 | 1.0-100.0 | 0.5 | 2.0 | 16.0 |
| Sulfadiazine | y = 2.46e^5^+1.18e^6^x | 0.9933 | 1.0-100.0 | 0.5 | 2.0 | 3.4 |
| Sulfatroxazole | y = 5.38e^5^+3.01e^6^x | 0.9908 | 1.0-100.0 | 0.5 | 2.0 | 8.3 |
| Sulfathiazole | y = -1.66e^4^+5.97e^5^x | 0.9960 | 1.0-100.0 | 0.5 | 2.0 | -7.2 |
| Sulfaethoxypyridazine | y = -2.83e^4^+1.24e^6^x | 0.9950 | 1.0-100.0 | 0.5 | 2.0 | 8.6 |
| Sulfapyrazole | y = -1.42e^5^+1.94e^6^x | 0.9939 | 1.0-100.0 | 0.5 | 2.0 | 12.1 |
| Fleroxacin | y = 9.87e^5^+2.26e^6^x | 0.9966 | 1.0-100.0 | 0.5 | 2.0 | -0.6 |
| Sparfloxacin | y = 5.08e^5^+2.96e^6^x | 0.9912 | 1.0-100.0 | 0.5 | 2.0 | 12.2 |
| Gatifloxacin | y = 8.83e^5^+2.53e^6^x | 0.9967 | 1.0-100.0 | 0.5 | 2.0 | 11.5 |
| Lomefloxacin | y = 4.92e^6^+2.26e^6^x | 0.9917 | 1.0-100.0 | 0.5 | 2.0 | 7.2 |
| Pefloxacin | y = 8.33e^6^+2.57e^6^x | 0.9886 | 1.0-100.0 | 0.5 | 2.0 | 1.3 |
| Ofloxacin | y = 3.62e^6^+2.45e^6^x | 0.9864 | 1.0-100.0 | 0.5 | 2.0 | 2.8 |
| Norfloxacin | y = 8.19e^5^+1.89e^6^x | 0.9911 | 1.0-100.0 | 0.5 | 2.0 | -42.3 |
| Danpfloxacin | y = 1.45e^6^+2.90e^6^x | 0.9973 | 1.0-100.0 | 0.5 | 2.0 | -6.2 |
| Difloxacin | y = 8.00e^5^+2.01e^6^x | 0.9938 | 1.0-100.0 | 0.5 | 2.0 | 7.8 |
| Enrofloxacin | y = 7.35e^7^+2.73e^6^x | 0.9950 | 1.0-100.0 | 0.5 | 2.0 | -68.2 |
| Flumequine | y = 3.09e^5^+4.79e^6^x | 0.9954 | 1.0-100.0 | 0.5 | 2.0 | 12.6 |
| Oxolinic acid | y = -2.43e^4^+1.38e^6^x | 0.9983 | 1.0-100.0 | 0.5 | 2.0 | 8.4 |
| Sarafloxacin | y = 5.83e^5^+1.71e^6^x | 0.9948 | 1.0-100.0 | 0.5 | 2.0 | 9.8 |
| Orbifloxacin | y = 1.03e^6^+2.42e^6^x | 0.9934 | 1.0-100.0 | 0.5 | 2.0 | 11.2 |
| Pipemidic acid | y = 1.15e^6^+1.78e^6^x | 0.9942 | 1.0-100.0 | 0.5 | 2.0 | 53.8 |
| Ciprofloxacin | y = 4.04e^6^+1.91e^6^x | 0.9921 | 1.0-100.0 | 0.5 | 2.0 | -1.5 |
| Marbofloxacin | y = 1.10e^6^+2.45e^6^x | 0.9969 | 1.0-100.0 | 0.5 | 2.0 | 11.6 |
| Nalidixic acid | y = 5.80e^5^+6.11e^6^x | 0.9955 | 1.0-100.0 | 0.5 | 2.0 | 9.5 |
| Cinoxacin | y = 5.84e^5^+3.31e^6^x | 0.9960 | 1.0-100.0 | 0.5 | 2.0 | 8.9 |
| Enoxacin | y = 8.13e^5^+1.24e^6^x | 0.9874 | 1.0-100.0 | 0.5 | 2.0 | 34.2 |
| Malachite green | y = -8.83e^5^+8.17e^6^x | 0.9967 | 1.0-100.0 | 0.5 | 2.0 | 4.9 |
| Crystal violet | y = -1.15e^6^+6.23e^6^x | 0.9960 | 1.0-100.0 | 0.5 | 2.0 | 0.1 |
| Diazepam | y = -3.20e^4^+3.93e^6^x | 0.9944 | 1.0-100.0 | 0.5 | 2.0 | 9.6 |
| Triazolam | y = 1.99e^5^+2.00e^6^x | 0.9948 | 1.0-100.0 | 0.5 | 2.0 | 8.8 |
| Lorazepam | y = -1.30e^5^+2.38e^5^x | 0.9979 | 1.0-100.0 | 0.5 | 2.0 | 9.8 |
| Nordiazepam | y = -8.71e^4^+1.01e^6^x | 0.9956 | 1.0-100.0 | 0.5 | 2.0 | 11.4 |
| Oxazepam | y = -1.28e^5^+2.97e^5^x | 0.9977 | 1.0-100.0 | 0.5 | 2.0 | 8.8 |
| Methaqualone | y = 8.74e^4^+6.14e^6^x | 0.9947 | 1.0-100.0 | 0.5 | 2.0 | 11.2 |
| Promethazine | y = 7.45e^5^+6.96e^6^x | 0.9971 | 1.0-100.0 | 0.5 | 2.0 | 13.3 |
| Temazepam | y = -2.30e^4^+8.36e^5^x | 0.9991 | 1.0-100.0 | 0.5 | 2.0 | 5.5 |
| Chlorpromazine | y = 6.59e^5^+4.86e^6^x | 0.9942 | 1.0-100.0 | 0.5 | 2.0 | 13.6 |
| Nitrazepam | y = -1.40e^5^+6.66e^5^x | 0.9960 | 1.0-100.0 | 0.5 | 2.0 | 13.1 |
| Estazolam | y = 1.37e^5^+1.68e^6^x | 0.9950 | 1.0-100.0 | 0.5 | 2.0 | 8.6 |
| Alprazolam | y = 4.50e^5^+2.41e^6^x | 0.9928 | 1.0-100.0 | 0.5 | 2.0 | 10.9 |
| Clenbuterol | y = 1.21e^6^+3.24e^6^x | 0.9953 | 1.0-100.0 | 0.5 | 2.0 | 12.0 |
| Cimaterol | y = -8.91e^4^+2.25e^6^x | 0.9957 | 1.0-100.0 | 0.5 | 2.0 | 34.2 |
| Bamethan | y = 9.01e^5^+1.89e^6^x | 0.9922 | 1.0-100.0 | 0.5 | 2.0 | 34.1 |
| Bambuterol | y = 1.65e^6^+5.51e^6^x | 0.9967 | 1.0-100.0 | 0.5 | 2.0 | 9.8 |
| Phenylethanolamine A | y = 4.29e^5^+3.25e^6^x | 0.9922 | 1.0-100.0 | 0.5 | 2.0 | 7.0 |
| Ractopamine | y = 1.29e^6^+2.84e^6^x | 0.9876 | 1.0-100.0 | 0.5 | 2.0 | 11.2 |
| Ritodrine | y = 1.44e^6^+3.23e^6^x | 0.9935 | 1.0-100.0 | 0.5 | 2.0 | 10.4 |
| Mabuterol | y = 1.20e^6^+4.27e^6^x | 0.9947 | 1.0-100.0 | 0.5 | 2.0 | 9.9 |
| Mapenterol | y = 6.77e^5^+4.70e^6^x | 0.9953 | 1.0-100.0 | 0.5 | 2.0 | 12.9 |
| Penbutolol | y = 6.95e^6^+9.03e^6^x | 0.9953 | 1.0-100.0 | 0.5 | 2.0 | 8.7 |
| Salmeterol | y = 1.20e^6^+2.33e^6^x | 0.9754 | 1.0-100.0 | 0.5 | 2.0 | 4.2 |
| Terbutaline | y = 4.94e^4^+1.67e^6^x | 0.9981 | 1.0-100.0 | 0.5 | 2.0 | 31.8 |
| Tulobuterol | y = 2.92e^6^+6.19e^6^x | 0.9946 | 1.0-100.0 | 0.5 | 2.0 | 11.7 |
| Cimbuterol | y = 1.92e^6^+3.49e^6^x | 0.9950 | 1.0-100.0 | 0.5 | 2.0 | 56.9 |
| Bromobuterol | y = 3.61e^5^+1.03e^6^x | 0.9929 | 1.0-100.0 | 0.5 | 2.0 | 10.9 |
| Isoxsuprine | y = 1.82e^6^+5.08e^6^x | 0.9926 | 1.0-100.0 | 0.5 | 2.0 | 12.0 |
| Isoetarine | y = 4.72e^5^+2.62e^6^x | 0.9936 | 1.0-100.0 | 0.5 | 2.0 | 32.6 |
| Clenproperol | y = 9.61e^5^+2.19e^6^x | 0.9946 | 1.0-100.0 | 0.5 | 2.0 | 9.5 |
| Clencyclohexerol | y = 1.43e^6^+1.40e^6^x | 0.9807 | 1.0-100.0 | 0.5 | 2.0 | 19.2 |
| Roxithromycin | y = -3.86e^4^+4.59e^5^x | 0.9971 | 1.0-100.0 | 0.5 | 2.0 | 4.5 |
| Clarithromycin | y = -1.56e^4^+1.19e^6^x | 0.9976 | 1.0-100.0 | 0.5 | 2.0 | 13.6 |
| Azithromycin | y = 9.34e^4^+3.41e^5^x | 0.9969 | 1.0-100.0 | 0.5 | 2.0 | 5.3 |
| Clindamycin | y = 1.99e^5^+6.30e^5^x | 0.9931 | 1.0-100.0 | 0.5 | 2.0 | 12.6 |
| Timicosin | y = -1.89e^4^+2.24e^5^x | 0.9990 | 1.0-100.0 | 0.5 | 2.0 | 0.0 |
| Tylosin | y = -1.43e^5^+1.39e^5^x | 0.9985 | 1.0-100.0 | 0.5 | 2.0 | 14.9 |
| Rifaximin | y = -4.33e^5^+7.53e^5^x | 0.9931 | 1.0-100.0 | 0.5 | 2.0 | -2.6 |
| Oleandomycin phosphate | y = -1.75e^3^+3.97e^5^x | 0.9950 | 1.0-100.0 | 0.5 | 2.0 | 10.4 |
| Albendazole | y = 2.93e^5^+3.75e^6^x | 0.9888 | 1.0-100.0 | 0.5 | 2.0 | 9.6 |
| Albendazole-2-aminosulfone | y = -3.43e^4^+4.77e^5^x | 0.9980 | 1.0-100.0 | 0.5 | 2.0 | 48.5 |
| Flubendazole | y = -6.57e^4^+1.23e^6^x | 0.9929 | 1.0-100.0 | 0.5 | 2.0 | 5.5 |
| Mebendazole | y = 1.15e^6^+1.52e^6^x | 0.9943 | 1.0-100.0 | 0.5 | 2.0 | 4.6 |
| Hydroxymebendazole | y = 5.69e^5^+2.56e^6^x | 0.9901 | 1.0-100.0 | 0.5 | 2.0 | 11.7 |
| Triclabendazole | y = -5.32e^5^+1.14e^6^x | 0.9956 | 1.0-100.0 | 0.5 | 2.0 | 31.2 |
| Oxibendazole | y = 2.37e^5^+6.03e^6^x | 0.9949 | 1.0-100.0 | 0.5 | 2.0 | 7.7 |
| Thiabendazole | y = 1.31e^6^+5.88e^6^x | 0.9931 | 1.0-100.0 | 0.5 | 2.0 | 5.8 |
| Cambendazol | y = 1.41e^5^+3.75e^6^x | 0.9941 | 1.0-100.0 | 0.5 | 2.0 | 4.6 |
| Oxfendazole | y = 8.95e^4^+1.26e^6^x | 0.9969 | 1.0-100.0 | 0.5 | 2.0 | 4.9 |
| Ciclobendazole | y = 3.99e^5^+2.55e^6^x | 0.9953 | 1.0-100.0 | 0.5 | 2.0 | 5.5 |
| Febantel | y = -1.06e^6^+5.01e^6^x | 0.9907 | 1.0-100.0 | 0.5 | 2.0 | 8.2 |
| Mebendazole-amine | y = 1.31e^6^+2.13e^6^x | 0.9820 | 1.0-100.0 | 0.5 | 2.0 | 12.4 |
| Albendazole sulfoxide | y = 7.87e^5^+1.60e^6^x | 0.9952 | 1.0-100.0 | 0.5 | 2.0 | -0.8 |
| Albendazole sulfone | y = 7.09e^4^+9.18e^5^x | 0.9948 | 1.0-100.0 | 0.5 | 2.0 | 9.7 |
| Levamisole | y = -6.06e^5^+7.06e^6^x | 0.9948 | 1.0-100.0 | 0.5 | 2.0 | 12.0 |
| Metronidazole | y = 3.81e^5^+1.22e^6^x | 0.9938 | 1.0-100.0 | 0.5 | 2.0 | 35.9 |
| Dimetridazole | y = 4.71e^5^+1.10e^6^x | 0.9997 | 1.0-100.0 | 0.5 | 2.0 | 15.5 |
| Ronidazole | y = -1.09e^5^+5.85e^5^x | 0.9929 | 1.0-100.0 | 0.5 | 2.0 | 18.0 |
| Tinidazole | y = -7.01e^5^.9+9.38e^5^x | 0.9966 | 1.0-100.0 | 0.5 | 2.0 | 29.7 |
| Ipronidazole | y = -5.01e^4^+1.15e^6^x | 0.9968 | 1.0-100.0 | 0.5 | 2.0 | 12.5 |
| Hydroxyipronidazole | y = 3.20e^5^+6.83e^5^x | 0.9936 | 1.0-100.0 | 0.5 | 2.0 | 13.0 |
| Secnidazole | y = -3.49e^4^+1.80e^6^x | 0.9911 | 1.0-100.0 | 0.5 | 2.0 | 16.8 |

Table S5 Recoveries of analytes in muscle tissue of grass carp spiked with different levels of target compounds (n=6)

| Target Compound | Recovery (%, n=6) | | | Intra-day RSD (%, n=6) | | | Inter-day RSD (%, n=3) | | |
| --- | --- | --- | --- | --- | --- | --- | --- | --- | --- |
|  | 2.0 μg·kg^-1^ | 5.0 μg·kg^-1^ | 16.0 μg·kg^-1^ | 2.0 μg·kg^-1^ | 5.0 μg·kg^-1^ | 16.0 μg·kg^-1^ | 2.0 μg·kg^-1^ | 5.0 μg·kg^-1^ | 16.0 μg·kg^-1^ |
| Tetracycline | 49.5 | 89.2 | 75.3 | 14.1 | 6.9 | 7.8 | 14.2 | 17.1 | 9.7 |
| Chlortetracycline | 49.1 | 91.1 | 78.0 | 21.0 | 7.9 | 9.3 | 19.7 | 13.7 | 11.3 |
| Oxytetracycline | 52.4 | 126.7 | 88.9 | 17.2 | 18.4 | 25.2 | 12.7 | 11.7 | 19.4 |
| Doxycycline | 73.0 | 113.9 | 83.8 | 12.0 | 14.5 | 11.6 | 14.7 | 15.0 | 11.9 |
| Trimethoprim | 90.3 | 88.2 | 83.9 | 4.9 | 6.1 | 5.7 | 18.0 | 8.5 | 5.4 |
| Sulfaphenazole | 98.0 | 61.5 | 121.1 | 11.0 | 17.0 | 10.6 | 13.6 | 15.1 | 13.0 |
| Sulfabemzamide | 64.1 | 65.3 | 84.7 | 15.3 | 9.6 | 2.6 | 18.3 | 18.8 | 6.4 |
| Sulfapyridine | 138.4 | 92.0 | 89.7 | 7.1 | 6.4 | 6.1 | 10.2 | 8.4 | 9.6 |
| Sulfameter | 83.1 | 74.0 | 87.3 | 9.7 | 10.7 | 5.2 | 12.8 | 10.1 | 9.0 |
| Sulfamoxol | 75.4 | 73.2 | 74.4 | 10.4 | 10.2 | 10.0 | 16.8 | 13.9 | 24.5 |
| Sulfamethazine | 84.2 | 71.2 | 89.9 | 8.1 | 10.1 | 6.6 | 9.8 | 8.4 | 10.6 |
| Sulfisoxazole | 73.9 | 62.8 | 92.9 | 12.6 | 12.8 | 6.2 | 13.0 | 16.4 | 5.7 |
| Sulfisomidine | 91.0 | 87.3 | 79.0 | 6.4 | 5.1 | 6.0 | 16.7 | 6.0 | 12.0 |
| Sulfamethoxazole | 77.4 | 65.9 | 96.9 | 13.2 | 11.9 | 3.4 | 14.7 | 16.2 | 6.9 |
| Sulfamerazine | 81.4 | 71.6 | 92.1 | 12.3 | 10.7 | 5.7 | 10.2 | 10.3 | 7.0 |
| Sulfamethizole | 76.2 | 68.5 | 85.4 | 11.7 | 12.2 | 8.4 | 11.9 | 11.8 | 12.1 |
| Sulfamehoxypyridazine | 78.5 | 65.6 | 92.6 | 9.2 | 11.9 | 6.1 | 9.7 | 13.5 | 4.9 |
| Sulfadimethoxine | 69.8 | 61.4 | 94.8 | 12.5 | 11.8 | 4.2 | 11.9 | 16.6 | 8.8 |
| Sulfamonomethoxine | 77.4 | 65.0 | 92.8 | 11.9 | 9.3 | 7.4 | 11.5 | 10.3 | 8.6 |
| Sulfaquinoxalin | 78.2 | 55.3 | 98.2 | 15.0 | 18.3 | 16.2 | 13.6 | 16.8 | 19.0 |
| Sulfadoxine | 81.9 | 67.5 | 96.2 | 12.4 | 11.7 | 5.0 | 9.5 | 12.5 | 5.0 |
| Sulfaclozone | 47.0 | 56.8 | 86.3 | 17.8 | 14.2 | 6.0 | 19.8 | 20.7 | 4.2 |
| Sulfachloropyridazine | 68.6 | 58.0 | 95.3 | 11.5 | 13.2 | 7.6 | 15.4 | 17.9 | 5.8 |
| Sulfadiazine | 103.4 | 67.5 | 106.0 | 10.3 | 15.3 | 12.6 | 9.8 | 17.7 | 12.4 |
| Sulfatroxazole | 81.4 | 65.2 | 94.9 | 11.0 | 12.5 | 5.6 | 10.0 | 11.4 | 6.9 |
| Sulfathiazole | 77.3 | 72.6 | 85.0 | 12.6 | 8.3 | 6.9 | 14.8 | 6.5 | 9.3 |
| Sulfaethoxypyridazine | 84.1 | 66.4 | 92.3 | 11.7 | 12.2 | 9.0 | 9.4 | 9.7 | 13.6 |
| Sulfapyrazole | 66.1 | 59.7 | 87.5 | 14.7 | 11.8 | 6.6 | 13.7 | 20.7 | 5.6 |
| Fleroxacin | 100.2 | 86.0 | 93.0 | 5.3 | 6.5 | 6.2 | 16.1 | 6.7 | 6.9 |
| Sparfloxacin | 83.4 | 82.4 | 84.7 | 4.2 | 5.5 | 2.9 | 14.0 | 6.6 | 7.9 |
| Gatifloxacin | 86.3 | 83.2 | 84.7 | 5.1 | 5.6 | 5.5 | 16.8 | 8.6 | 5.7 |
| Lomefloxacin | 104.0 | 89.0 | 78.2 | 6.5 | 4.2 | 6.0 | 16.8 | 10.4 | 5.9 |
| Pefloxacin | 109.4 | 94.4 | 90.3 | 3.5 | 4.7 | 6.1 | 18.6 | 14.8 | 8.4 |
| Ofloxacin | 93.5 | 92.6 | 84.1 | 10.2 | 4.4 | 5.9 | 19.6 | 12.1 | 6.3 |
| Norfloxacin | 79.0 | 88.8 | 76.2 | 6.5 | 5.4 | 6.0 | 18.0 | 18.4 | 9.3 |
| Danpfloxacin | 87.6 | 87.7 | 82.8 | 3.8 | 6.3 | 7.0 | 19.9 | 16.4 | 9.5 |
| Difloxacin | 100.8 | 77.5 | 104.9 | 8.9 | 8.7 | 6.3 | 13.1 | 7.9 | 4.7 |
| Enrofloxacin | 137.3 | 121.6 | 101.3 | 5.7 | 5.9 | 6.5 | 14.7 | 15.8 | 14.3 |
| Flumequine | 80.3 | 65.7 | 101.4 | 12.8 | 13.2 | 2.3 | 10.4 | 17.5 | 6.2 |
| Oxolinic acid | 82.1 | 68.6 | 99.3 | 11.8 | 6.6 | 3.6 | 9.7 | 13.1 | 8.4 |
| Sarafloxacin | 83.9 | 78.9 | 88.9 | 5.3 | 7.3 | 6.2 | 13.8 | 6.3 | 5.4 |
| Orbifloxacin | 87.5 | 82.9 | 87.1 | 5.7 | 7.2 | 3.7 | 16.3 | 6.6 | 8.7 |
| Pipemidic acid | 66.6 | 88.4 | 67.0 | 3.2 | 7.7 | 7.1 | 20.5 | 18.9 | 9.7 |
| Ciprofloxacin | 137.3 | 107.5 | 78.9 | 4.0 | 4.4 | 6.9 | 7.6 | 5.6 | 4.0 |
| Marbofloxacin | 92.0 | 89.0 | 86.7 | 4.0 | 6.5 | 6.3 | 19.6 | 12.7 | 7.7 |
| Nalidixic acid | 85.5 | 68.1 | 101.7 | 12.3 | 12.4 | 2.4 | 11.9 | 16.5 | 7.1 |
| Cinoxacin | 84.4 | 70.6 | 100.2 | 10.1 | 10.2 | 3.4 | 8.9 | 11.0 | 9.1 |
| Enoxacin | 70.5 | 86.8 | 73.5 | 4.8 | 7.0 | 7.8 | 17.4 | 19.2 | 9.0 |
| Malachite green | 35.3 | 67.2 | 52.2 | 18.0 | 11.8 | 6.3 | 17.2 | 23.7 | 6.0 |
| Crystal violet | 54.8 | 69.6 | 76.4 | 17.8 | 17.5 | 4.5 | 18.7 | 19.0 | 5.2 |
| Diazepam | 76.2 | 69.7 | 85.1 | 15.3 | 16.1 | 4.7 | 13.7 | 21.8 | 5.3 |
| Triazolam | 81.9 | 69.9 | 101.2 | 10.7 | 13.4 | 2.8 | 11.7 | 16.0 | 7.6 |
| Lorazepam | 49.9 | 60.3 | 95.9 | 17.6 | 18.1 | 3.8 | 19.3 | 17.0 | 8.8 |
| Nordiazepam | 63.8 | 69.1 | 80.8 | 14.6 | 16.9 | 3.6 | 13.1 | 18.5 | 3.9 |
| Oxazepam | 58.4 | 64.5 | 90.4 | 19.9 | 15.6 | 2.1 | 17.5 | 18.3 | 6.8 |
| Methaqualone | 71.5 | 68.1 | 89.0 | 13.7 | 14.6 | 5.0 | 13.3 | 20.6 | 4.7 |
| Promethazine | 64.7 | 73.0 | 74.9 | 12.2 | 10.2 | 5.4 | 11.8 | 20.5 | 4.9 |
| Temazepam | 73.9 | 56.8 | 92.3 | 16.7 | 14.7 | 4.4 | 15.4 | 21.4 | 8.5 |
| Chlorpromazine | 56.0 | 72.7 | 66.8 | 12.7 | 12.3 | 3.2 | 12.0 | 15.4 | 1.9 |
| Nitrazepam | 63.4 | 67.4 | 90.3 | 17.0 | 14.7 | 4.9 | 15.2 | 20.7 | 4.4 |
| Estazolam | 84.8 | 69.3 | 104.2 | 8.8 | 12.4 | 2.2 | 11.4 | 15.2 | 7.4 |
| Alprazolam | 82.3 | 71.5 | 101.4 | 9.8 | 11.3 | 2.4 | 9.8 | 14.9 | 6.5 |
| Clenbuterol | 88.9 | 77.9 | 93.8 | 7.2 | 7.5 | 2.6 | 11.8 | 9.0 | 8.0 |
| Cimaterol | 78.8 | 83.2 | 81.6 | 6.0 | 7.5 | 7.0 | 17.0 | 19.3 | 19.0 |
| Bamethan | 88.3 | 92.8 | 74.2 | 5.5 | 6.2 | 4.7 | 20.1 | 9.2 | 9.1 |
| Bambuterol | 98.6 | 86.8 | 93.4 | 5.8 | 5.4 | 5.4 | 19.5 | 8.5 | 7.0 |
| Phenylethanolamine A | 71.9 | 66.1 | 91.7 | 10.5 | 15.3 | 3.1 | 11.8 | 13.5 | 8.2 |
| Ractopamine | 77.6 | 78.1 | 84.8 | 8.3 | 7.5 | 3.0 | 13.2 | 5.5 | 7.7 |
| Ritodrine | 76.3 | 80.6 | 81.8 | 7.0 | 6.3 | 1.9 | 11.2 | 7.1 | 6.6 |
| Mabuterol | 87.4 | 78.0 | 93.7 | 7.8 | 8.6 | 4.3 | 12.3 | 11.4 | 1.8 |
| Mapenterol | 87.3 | 71.6 | 100.0 | 10.9 | 13.0 | 3.6 | 10.1 | 14.8 | 7.5 |
| Penbutolol | 68.9 | 76.2 | 76.2 | 14.7 | 13.4 | 4.1 | 13.0 | 21.6 | 2.9 |
| Salmeterol | 68.2 | 73.6 | 82.1 | 12.2 | 11.5 | 2.0 | 12.6 | 22.0 | 6.2 |
| Terbutaline | 58.8 | 78.5 | 83.6 | 9.2 | 20.4 | 10.2 | 19.9 | 18.0 | 15.8 |
| Tulobuterol | 92.5 | 84.8 | 89.4 | 4.9 | 7.1 | 5.2 | 14.8 | 8.1 | 4.4 |
| Cimbuterol | 88.9 | 86.4 | 83.3 | 5.5 | 5.9 | 5.9 | 16.1 | 6.8 | 4.7 |
| Bromobuterol | 84.4 | 79.0 | 88.6 | 6.5 | 9.6 | 5.0 | 13.0 | 10.5 | 3.2 |
| Isoxsuprine | 84.8 | 92.8 | 85.7 | 7.5 | 7.2 | 4.1 | 11.9 | 10.4 | 6.9 |
| Isoetarine | 65.5 | 62.7 | 78.4 | 12.2 | 18.6 | 21.4 | 18.8 | 19.0 | 20.1 |
| Clenproperol | 88.0 | 82.0 | 84.8 | 6.2 | 6.6 | 5.8 | 13.9 | 8.0 | 4.2 |
| Clencyclohexerol | 78.4 | 89.2 | 71.5 | 4.4 | 5.5 | 5.2 | 15.1 | 7.7 | 3.4 |
| Roxithromycin | 75.6 | 73.0 | 89.4 | 10.3 | 10.2 | 6.3 | 14.4 | 13.0 | 4.8 |
| Clarithromycin | 90.2 | 95.9 | 90.3 | 6.6 | 9.7 | 4.0 | 12.8 | 9.8 | 9.3 |
| Azithromycin | 99.7 | 93.6 | 88.9 | 5.7 | 7.5 | 6.8 | 31.8 | 19.9 | 15.2 |
| Clindamycin | 65.1 | 83.8 | 86.4 | 7.6 | 7.1 | 9.5 | 13.7 | 16.8 | 13.0 |
| Timicosin | 88.5 | 88.9 | 92.5 | 11.6 | 6.7 | 6.9 | 18.7 | 20.5 | 8.2 |
| Tylosin | 73.3 | 82.1 | 72.8 | 19.4 | 12.4 | 15.4 | 17.3 | 16.5 | 13.0 |
| Rifaximin | 67.7 | 55.3 | 84.1 | 14.8 | 19.7 | 7.8 | 14.5 | 25.4 | 8.3 |
| Oleandomycin phosphate | 97.0 | 83.9 | 102.0 | 6.4 | 7.5 | 2.3 | 17.3 | 4.3 | 7.6 |
| Albendazole | 47.2 | 68.4 | 57.0 | 15.8 | 16.9 | 2.6 | 19.3 | 13.8 | 14.5 |
| Albendazole-2-aminosulfone | 86.4 | 80.0 | 92.6 | 4.7 | 8.2 | 8.0 | 15.7 | 8.2 | 6.6 |
| Flubendazole | 67.9 | 65.8 | 74.6 | 15.3 | 18.3 | 5.0 | 14.2 | 20.8 | 5.2 |
| Mebendazole | 120.0 | 93.7 | 80.3 | 6.9 | 12.1 | 3.9 | 22.4 | 20.5 | 10.7 |
| Hydroxymebendazole | 89.5 | 70.4 | 99.5 | 11.8 | 14.8 | 6.4 | 9.4 | 12.2 | 5.4 |
| Triclabendazole | 57.7 | 54.6 | 70.5 | 18.6 | 21.3 | 7.4 | 18.5 | 22.0 | 12.2 |
| Oxibendazole | 62.2 | 71.6 | 72.8 | 14.5 | 14.3 | 2.3 | 16.7 | 14.3 | 8.1 |
| Thiabendazole | 87.8 | 75.5 | 92.6 | 8.9 | 9.6 | 4.6 | 9.0 | 11.2 | 2.6 |
| Cambendazol | 79.4 | 67.7 | 98.3 | 10.8 | 12.6 | 2.7 | 10.4 | 18.2 | 6.9 |
| Oxfendazole | 96.9 | 66.8 | 104.5 | 14.0 | 18.5 | 8.9 | 12.2 | 15.2 | 7.3 |
| Ciclobendazole | 84.0 | 65.5 | 95.4 | 12.8 | 17.0 | 9.3 | 18.2 | 15.8 | 16.0 |
| Febantel | 61.0 | 60.8 | 82.1 | 16.7 | 18.0 | 6.8 | 10.0 | 15.0 | 7.1 |
| Mebendazole-amine | 66.9 | 80.3 | 78.0 | 7.9 | 7.7 | 3.0 | 15.4 | 22.2 | 8.0 |
| Albendazole sulfoxide | 92.4 | 97.6 | 93.6 | 8.7 | 8.7 | 6.0 | 16.8 | 17.8 | 11.1 |
| Albendazole sulfone | 105.3 | 60.6 | 116.1 | 13.5 | 26.1 | 17.0 | 11.8 | 8.4 | 9.8 |
| Levamisole | 75.3 | 72.4 | 92.4 | 9.2 | 10.3 | 5.3 | 14.0 | 14.4 | 15.8 |
| Metronidazole | 91.1 | 76.5 | 94.2 | 9.1 | 6.6 | 3.1 | 13.2 | 5.6 | 7.8 |
| Dimetridazole | 81.2 | 67.4 | 105.4 | 8.0 | 9.0 | 2.0 | 17.7 | 10.8 | 7.0 |
| Ronidazole | 83.3 | 74.6 | 101.2 | 7.0 | 7.2 | 6.5 | 8.7 | 12.9 | 8.2 |
| Tinidazole | 82.3 | 86.5 | 95.1 | 9.8 | 12.4 | 4.7 | 12.2 | 7.4 | 5.9 |
| Ipronidazole | 77.6 | 67.0 | 100.7 | 12.7 | 12.7 | 2.5 | 9.2 | 10.8 | 9.6 |
| Hydroxyipronidazole | 102.4 | 82.3 | 106.5 | 9.6 | 10.4 | 3.5 | 11.4 | 16.3 | 6.3 |
| Secnidazole | 99.7 | 82.2 | 99.9 | 8.4 | 6.8 | 6.4 | 13.0 | 9.3 | 4.9 |

Table S6 Comparison of different single-step cleanup methods for quick screening of drug residues

| Matrix | Analytes | Cleanup method | Detector | Whether the extract in tube could all pass through the r-DSPE layer | Number of samples in parallel manually | Recoveries | LOQs (μg·kg^-1^) | Analysis time | Reference |
| --- | --- | --- | --- | --- | --- | --- | --- | --- | --- |
| Apple, etc. | 350 Pesticide Residues | m-PFC cleanup method | GC-Orbitrap HRMS | Yes | 1 | 72.8 - 122.4% | 1.0-10.0 | No reported | (Meng et al., 2021) |
| Tea | 20 pesticides and metabolites residues | Sin-QuEChERS column | HPLC-Q-Orbitrap HRMS | No | 1 | 74 - 111% | 2.0-10.0 | No reported | (Huang et al., 2021) |
| Fish | 112 veterinary drugs | Filter-press single-step cleanup column | HPLC-Q-Orbitrap HRMS | Yes | 5 | 35.3 - 138.4 % | 2.0 | ≈2 min/5 samples | Our work |

Table S7 Detected analytes in real samples with filter-press clean-up method (μg·kg^-1^)

| Sample No. | Real sample | Compounds | | | | | |
| --- | --- | --- | --- | --- | --- | --- | --- |
|  |  | Oxytetracycline | Pefloxacin | Ciprofloxacin | Enrofloxacin | Azithromycin | Mebendazole |
| 1 | Channel catfish | - | - | 3.4 | 159.6 | - | - |
| 2 | Channel catfish | - | - | - | 33.6 | - | - |
| 3 | Carp | - | 3.6 | - | 3.8 | - | - |
| 4 | Channel catfish | - | 3.5 | - | 5.7 | - | - |
| 5 | Carp | - | 7.9 | - | - | - | 5.6 |
| 6 | Carp | - | 7.5 | - | - | - | - |
| 7 | Carp | - | - | - | 8.2 | - | - |
| 8 | Channel catfish | - | - | 2.2 | 136.5 | - | - |
| 9 | Channel catfish | - | - | 2.5 | 173.7 | - | - |
| 10 | Carp | - | - | - | 12.1 | - | - |
| 11 | Channel catfish | - | - | 15.6 | 198.1 | - | - |
| 12 | Carp | - | - | - | 6.1 | - | - |
| 13 | Garss carp | 3.2 | - | - | 9.4 | - | - |
| 14 | Garss carp | - | - | - | 3.4 | - | - |
| 15 | Yellow-head catfish meat | - | - | - | 5.4 | - | - |
| 16 | Garss carp | - | 2.6 | - | - | - | - |
| 17 | Channel catfish | - | - | - | 53.0 | 86.1 | - |
| 18 | Channel catfish | - | - | - | 18.5 | 25.1 | - |
| 19 | Channel catfish | - | 4.1 | - | 25.8 | 33.0 | - |
| 20 | Channel catfish | - | - | - | 8.7 | 10.8 | - |
| 21 | Channel catfish | - | - | - | 5.8 | - | - |
| 22 | Channel catfish | - | - | - | 6.5 | - | - |
| 23 | Channel catfish | - | - | - | 16.7 | - | - |
| 24 | Channel catfish | - | - | - | 8.0 | - | - |

“-”under LOQ or not detected.
